# Asthma diagnosis and treatment – 1006. Perillae semen abolished allergic asthmatic response in murine model

**DOI:** 10.1186/1939-4551-6-S1-P6

**Published:** 2013-04-23

**Authors:** Mi-Kyeong Kim, Tae Young Yoon, Byungkwon Choi

**Affiliations:** 1 Internal Medicine, Subdivision of Allergy, Chungbuk National University, Cheongjoo, South Korea; 2 Dermatology, Chubgbuk National University, Cheongjoo, South Korea; 3 Joongwon Dang, Cheongjoo, South Korea

## Background

Many inflammatory cells and cytokines play main role in allergic reaction, such as Th2 lymphocyte, mast cell, eosinophils, and interleukins such as IL-4,5,13. So those kinds of cells and cytokines would be the therapeutic targets in allergic inflammation. IL-10 regulates an allergic inflammation and makes tolerance, so it is one of good therapeutic modalities. Herb N(Perrilae semen) has been used as a medicine for anti-cough and other chest symptoms in our ancient medicine. Chemical components of Perillae semen and physico-chemical properties of Perillae semen oil were analyzed for the use as an edible oil. The proximate compositions of Perillae semen were 7.5% moisture, 33.2% crude fat, 16.3% crude protein, 2.8% crude ash, 6.5% crude fiber, and 33.7% nitrogen free extract. The major amino acids of Perillae semen were glutamic acid(66.9mg%), aspartic acid (32.5mg%), histidine(21.6mg%), and phenylaanine (20.1mg%). The ratio of essential/total amino acid was 41.3%. The physico-chemical properties of the seed oil were 0.915 specific gravity, 1.4808 refractive index, 3.6 acid value, 181.7 iodine value, and 194.0 saponification value. Composition of major lipid of the oil fractionated by silicic acid chromatography was 94.2% neutral lipids and 5.8% polar lioids. The major fatty acids of the oil were linolenic, linoleic and oleic acid. Here in this study, we tried to find the anti-asthmatic effects and its mechanism of herb N in murine asthma model.

## Methods

The effects of herb N were evaluated by antibodies such as OA-IgE, Penh(enhanced pause, OMP-3000) measured by methacholine. and cytokines IL-4 and IL-5, INF-gamma and IL-10 by RT-PCR.

## Results

Perrilae Semen significant abolished allergic asthmatic response. Infiltrated cells changed to small lymphocytes only, whose total number was 1/50 of allergic mice model in BAL fluid. Penh(enhanced pause) measured by methacholine challenge markedly decreased. IL-4 and 5 by RT-PCR were decreased, but INF-gamma and IL-10 increased.

## Conclusions

Perrilae Semen shifted immune response from Th2 to immune tolerance through the generationof IL-10. This will be the new therapeutic candidate of allergic disease leading to the tolerance.

